# Biomechanical consequences of anterior root detachment of the lateral meniscus and its reinsertion

**DOI:** 10.1038/s41598-022-10229-5

**Published:** 2022-04-13

**Authors:** Alejandro Espejo-Reina, Maria Prado-Novoa, Alejandro Espejo-Baena, Alejandro Peña-Trabalon, Ana Perez-Blanca

**Affiliations:** 1 Hospital Vithas Malaga, Malaga, Spain; 2 Clinical Biomechanics of Andalucia Laboratory, University of Malaga, Malaga, Spain; 3 Laboratory of Clinical Biomechanics, Department of Mechanical Engineering, Universidad de Málaga, Andalucía Tech, Málaga, Spain

**Keywords:** Biomedical engineering, Mechanical engineering, Translational research, Cartilage

## Abstract

Treatment of posterior meniscal roots tears evolved after biomechanical evidence of increased pressures on the tibiofemoral cartilage produced by this lesion and the subsequent accelerated development of arthritis or osteonecrosis observed clinically. However, little is known about the consequences of the detachment of the anterior roots. This in-vitro study analyzes the biomechanical changes in the tibiofemoral joint caused by avulsion of the anterior root of the lateral meniscus. The effectiveness of surgical root re-insertion to restore the pre-injured conditions is also evaluated. Using cadaveric knees at flexion angles from 0° to 90°, results show that the lesion significantly reduces the contact area and raises the pressure on the tibiofemoral cartilage of the injured compartment at all angles. Said modifications become larger at low flexion angles, which are the most frequent positions adopted by the knee in daily and sports activities, where they result similar to total meniscectomy. In-situ repair partially restores the contact biomechanics. Consequently, careful attention should be paid to proper diagnosis and treatment of detached anterior roots since the observed altered knee contact might induce similar degenerative problems in the cartilage as with completely detached posterior roots.

## Introduction

Integrity of the posterior meniscal roots is crucial to preserve meniscal function. The effects of an avulsion of the posterior roots have been well studied in recent years^1–6^. Biomechanical consequences of their detachment can be similar to those of a total meniscectomy^1,3,6^, leading to an increase in pressure on the affected compartment, and subsequently, rapid development of arthritis^7^ or osteonecrosis^8^. Root reinsertion applying surgical treatments promotes recovery towards the pre-injury biomechanics^1,2^.

Lesions of the anterior roots has received much less attention. Although it could be attributed to a low reported incidence compared with lesions of the posterior roots, there is a shortfall of comprehensive analyses addressing the incidence rate of anterior root tears. Recently, injuries to the anterior root of the lateral meniscus (ARLM) have been reported concomitant with some tibial fractures^7,9^ or in the setting of anterior cruciate ligament (ACL) reconstruction^10,11^, where a high risk of iatrogenic damage to the root during tunnel reaming exists due to the proximity of the root insertion to the tibial footprint of the ACL^12,13^, which occurred in up to 18% of the interventions in a study of Asian women^13^. After anterior meniscal root tears, early occurrence of osteoarthritis has been suggested in an animal model^14^. Specifically, in porcine models, histopathological changes in the cartilage (a hallmark of OA) from 1 month after surgical detachment of the anterior horn of the medial meniscus were reported^15–17^ as well as significant cartilage wear which became progressively more evident at 3 and 6 months^15,16^. Chondral lesions were also identified in a small series of humans after chronic anterior root tears^18^, indicating that disinsertion of the ARLM might have deleterious consequences. When a torn anterior root is found in the clinical context, the surgeon must choose the best treatment to apply. However, regarding ARLM avulsion a decision protocol is lacking and biomechanical studies are still needed to fully understand the effects of the lesion on the biomechanics of the knee and to assess the effectiveness of the repair.

The objective of this study was to analyze the consequences on the tibiofemoral contact mechanics of the human knee of an avulsion of the ARLM and its repair with an in-situ fixation technique. Our hypotheses were that the avulsion of the ARLM modifies tibiofemoral contact, increasing the pressure on the articular cartilage and that the repair of such injuries restores the biomechanical behavior of the knee.

## Methods

After approval by the Ethics Committee for Experimentation of the University of Málaga, nine frozen cadaveric human knees were initially included in the study (5 men and 4 women; mean age, 84 years; range 68–91 years). Specimens were provided by a specialized company that obtained informed consent from donor or next of kin. One specimen was discarded because an anomalous geometry of the external tibial compartment was observed after disarticulation at the end of its test. Thus, the final sample size was n = 8. The knees were suplied by a company (Ekokoes Tecnología y Servicios SL, Valencia, Spain) specialized in providing cadaveric specimens for educational, surgical training, or research purposes, and all experiments were performed in accordance with relevant guidelines and regulations.

One day before the test, the specimen was left at room temperature, wrapped in dampened gauze. Once thawed, the knee was dissected up to its capsular plane and visually inspected via an open arthrotomy incision for no previous pathologies, with special attention to the integrity of menisci and principal knee ligaments and no chondral injuries (the absence of pathologies was rechecked at the end of the test by disarticulating the joint up to the intraarticular surface was exposed). The arthrotomy was left open for the rest of the testing procedure. Next, the bones were cut to approximately 150 mm from the joint, and the distal ends of the bones were potted with epoxy resin in rectangular wooden receptacles while keeping the tibial and femoral axis at 90° to the bottom plane of the containers.

To characterize tibiofemoral contact pressure, pressure sensors were used (K-scan 4000, Tekscan Inc., Boston, MA) that consisted of two sections, each with an area 27.9 × 33 mm^2^ and a spatial resolution of 62 sensels/cm^2^. A new sensor was used for each specimen. Four tabs incorporated in the sensor were reinforced with adhesive tape before applying sutures that were used to guide sensor insertion. On the test day, immediately before use, the sensor was preconditioned by applying 5 cycles of 1000 N, and then a 3-point law calibration was performed before its insertion, following the manufacturer’s protocol.

**Figure 1:**
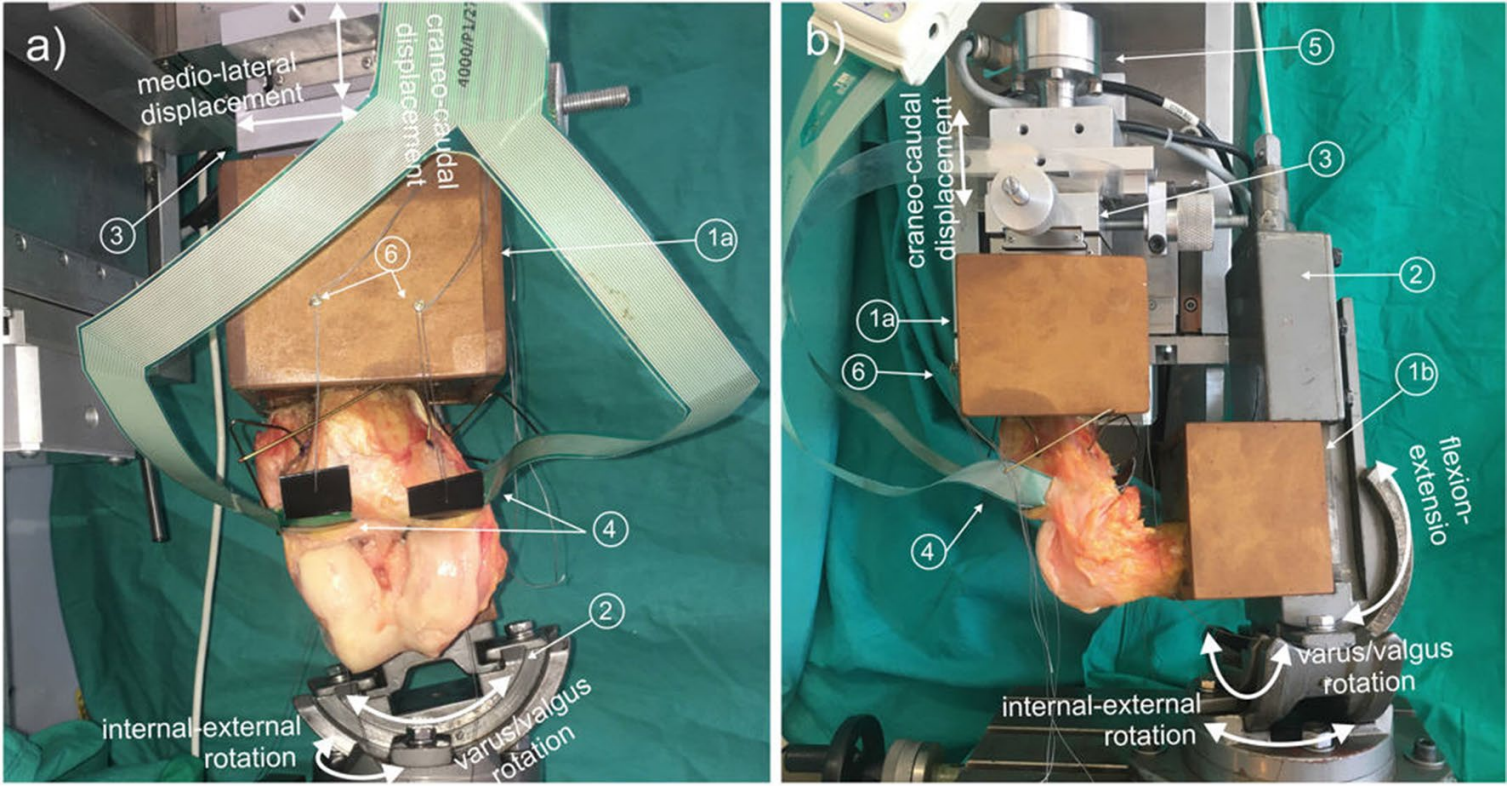
Experimental setup: (**a**) frontal and (**b**) lateral view showing the testing machine with a right knee specimen mounted at 90° flexion. White arrows highlight the machine elements that enable the movements associated with the degrees of freedom of the experiment. (**a**) Tibial container; (**b**) femoral container; 2: clamp that permits three-dimensional rotation used to fix the femur; 3: actuator of the machine with linear ball guides interposed; 4: pressure sensor; 5: load cell; 6: screws to fix the sensor to the tibial container.

Next, an orthopedic surgeon from our team performed all the preparations and surgical simulations. First, the coronary ligament was sectioned only as strictly necessary to introduce the sensor between each meniscus and the tibial articular surface, ensuring that the meniscal and knee ligaments were not damaged. The anterior intermeniscal ligament was also sectioned to homogenize the sample because this structure is not present in all the specimens, and when it is found, its characteristics are highly variable and its unclear role may influence knee biomechanics^19,20^. As the rectangular sections of the sensor did not exactly match the intraarticular surfaces, they were specifically placed to maximize coverage of the contact areas for each meniscal condition and flexion angle, as assessed by the computer image of the sensor with the knee flexed under manual compression. Once positioned, the sutures used for insertion were tied to screws attached to the tibia container to minimize sensor movement during loading (Fig. 1).

**Figure 2:**
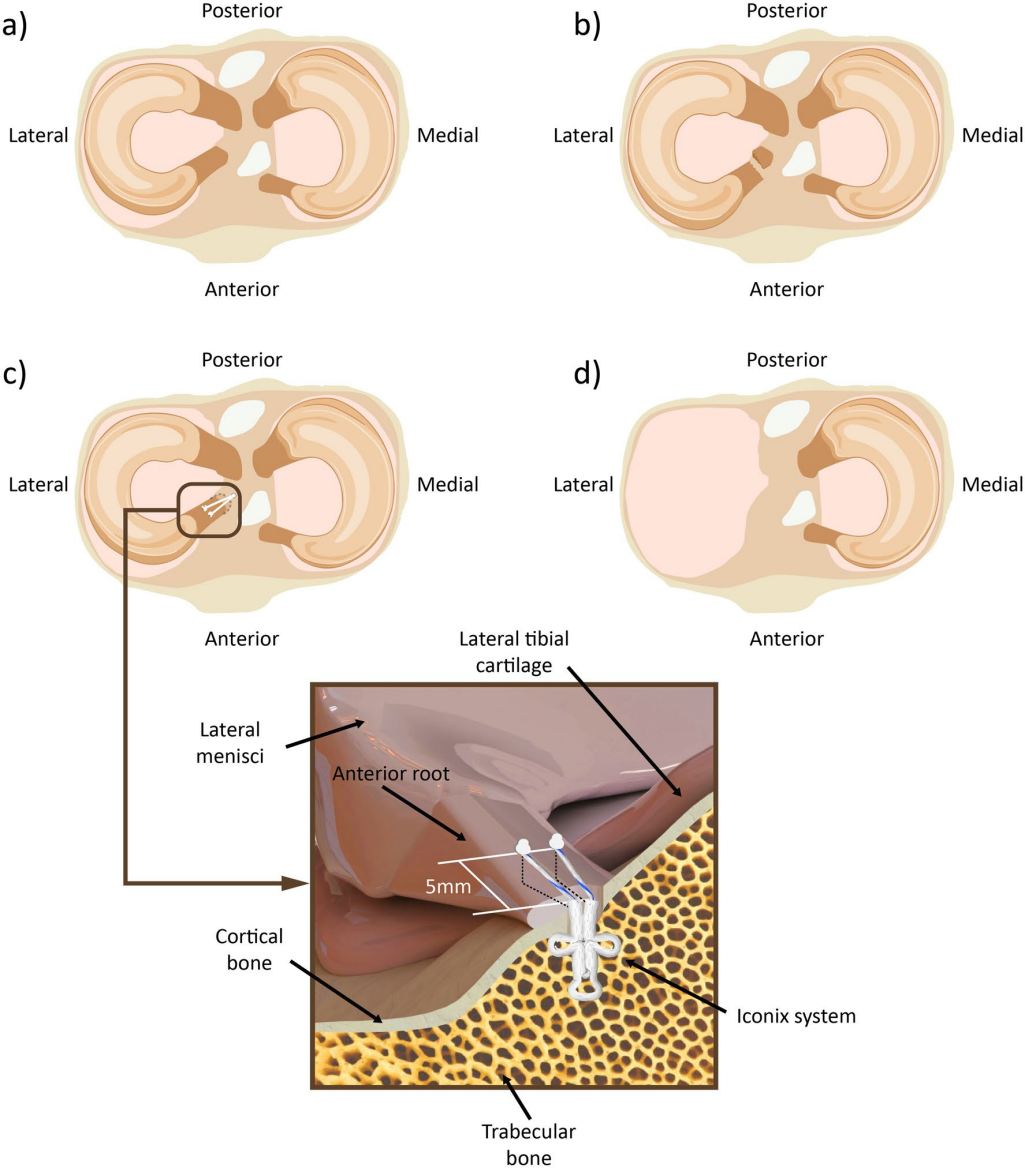
Schematics of the lateral meniscus conditions tested: (**a**) intact meniscus, (**b**) anterior root detachment, (**c**) In-situ repair, (**d**) meniscectomy. The detail shows a magnified representation of the repair technique.

Each knee was tested in four different conditions of the lateral meniscus (Fig. 2) in the following order: (1) intact (reference condition); (2) injured: detachment of the ARLM; (3) repair: simulated surgical reinsertion of the ARLM; and (4) total meniscectomy. For every condition, the specimen was tested at four flexion angles in the following order: 0°, 30°, 60°, and 90°, where 0° corresponded to full extension. These flexion angles are consistent with those used in previous studies on the human meniscus^1,3,6,21,22^.

Once the tests were completed in the intact condition, the ARLM was transected with a scalpel. When the tests with the simulated lesion were completed, the repair was carried out using a suture anchor (Iconix® 2.3 mm, two threads; Stryker, Greenwood Village, CO, USA) for in-situ fixation (Fig. 2). After inserting the anchor in the anatomic footprint of the ARLM articular tibial surface according to the manufacturer’s instructions, proper stability of the implant was checked by pulling on the thread tails. Subsequently, with the aid of a needle, two sutures were applied by passing an end of each thread through the meniscus and then knotting both tails together. The suture locations were approximately 5 mm from the anterior edge of the lateral meniscus and separated by 5 mm from each other. Finally, the lateral meniscus was removed to test for the last condition.

### Biomechanical testing

A custom uniaxial traction/compression testing machine^23^ was used for the tests (Fig. 1). The bottom of the tibial container was coupled to the actuator of the machine with the tibial axis aligned in the loading direction. Two perpendicular flat linear ball guides (BWU 60–60, IKO, Tokyo, Japan) were placed between the container and actuator to free the mediolateral and anteroposterior tibial displacements (Fig. 1). The container of the femur was connected to the base of the testing machine with a clamp that fixed the flexion angle and allowed varus-valgus and internal–external rotations.

**Figure 3:**
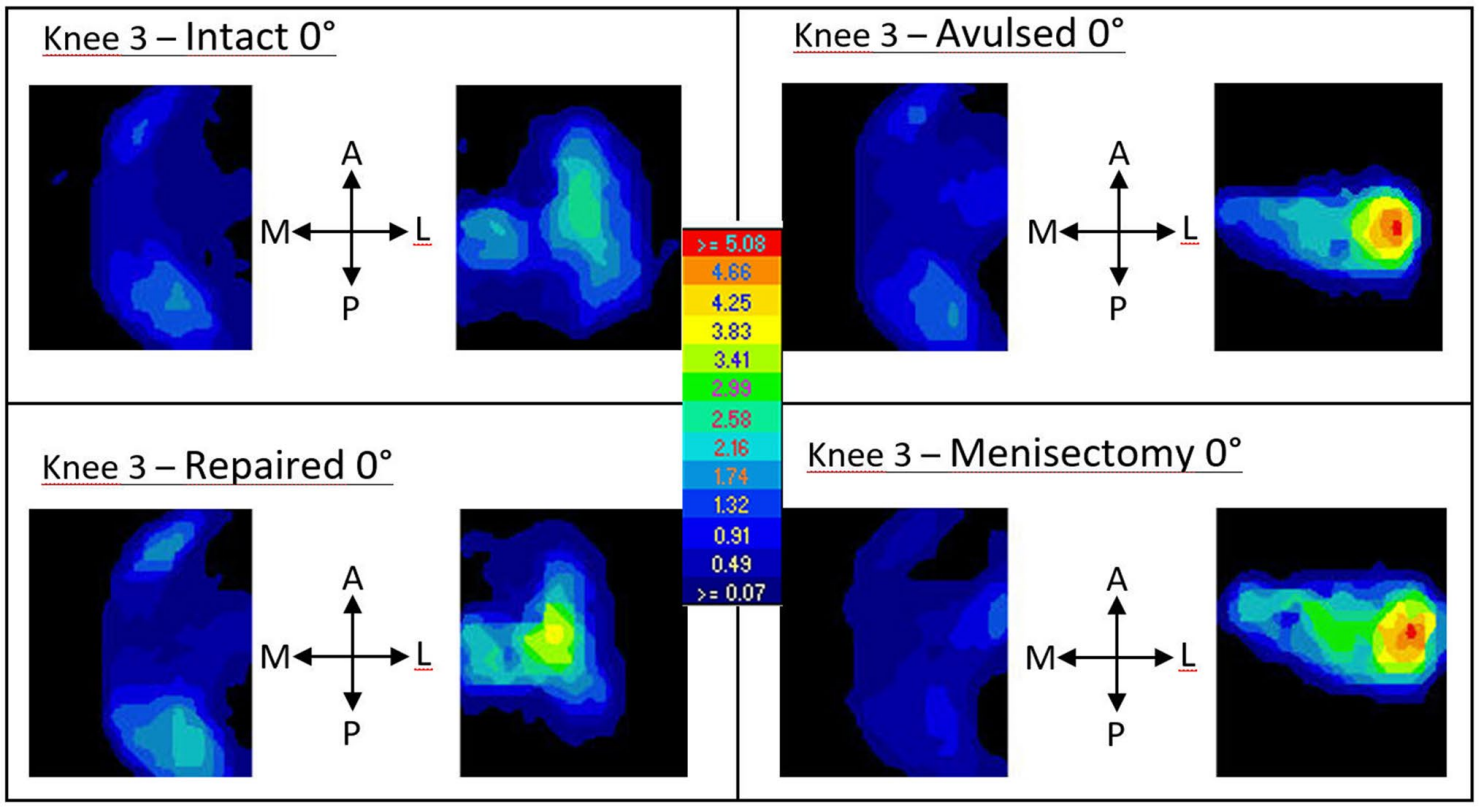
Representative distribution of pressures in the lateral and medial compartments. The four lateral meniscus conditions tested are represented for specimen n° 3 at 0° of flexion: anterior root avulsion of the lateral compartment resulted in a more concentrated distribution of pressures in the injured side, with steeper gradients. These effects were similar to meniscectomy. With repair, pressure distribution tended to recovered that of the intact condition.

For each flexion angle and meniscal condition, firstly, a slowly increasing compressive load from 0 to 100 N was applied at 0.1 mm/s while permitting natural alignment of the specimen. Once this load level was reached, anteroposterior displacement was blocked , as in previous works^24^,to avoid later instability of the knee owing to the absence of muscles^25^ (as was evidenced in our pilot tests, specially at the higher flexion angles). Immediately after, axial compression was increased at 1 mm/s from 100 N until it reached 1000 N. Then, this load was held for 1 min to let stabilize the signal from the pressure sensors (Fig. 3) and to minimize the possible variations in these recordings between testing conditions due to viscoelastic effects. At this point the contact pressure was recorded. The 1000 N axial compression was selected to facilitate comparisons with previous studies on the human meniscus^1,3,6,21,22^, although the value is greater than that expected in the immediate postoperative period.

The force was recorded by a class 1 load cell of 2kN rating (HBM, Darmstadt, Germany) located between the container of the tibia and the machine actuator (Fig. 1). The data were exported to a text file and was processed using MatLab®v.R2019b (The Mathworks Inc., Massachusetts, USA). Some studies have reported a decrease in the pressure magnitude measured by Tekscan after the application of several dynamic loading cycles^3,26,27^. In our pilot test, we also observed a linear decline over time of the load recorded after 16 measuring cycles per sensor. As in previous works^18^, to correct the discrepancy between applied load and total force provided by the sensor (computed as summation of sensel pressure x area), the data acquired in every test case was normalized. To exclude signal noise and other confounding values, such as those generated by the presence of wrinkles in the sensor, from the analyses, only pressures greater than 0.07 MPa were considered^28^. The following values were computed for each compartment at every combination of lateral meniscus condition and flexión angle: contact area, mean pressure, and peak pressure.

### Statistical analyses

To control the inter-specimen variability due to differences in shape, size, or natural alignment, the parameters related to the pressure and size of the contact area were normalized by calculating their ratios relative to the same parameter in the intact condition at the same flexion angle.

To assess for differences between testing conditions, non-parametric tests were applied as the most appropriate for small samples in which it is not known whether the population distribution is normal. The resulting ratios of the contact area, mean pressure, and peak pressure for each testing condition were compared with a value of 1 to assess for any variation relative to the reference condition using a Wilcoxon signed-rank test. Differences between treatments were evaluated using Friedman’s analysis of variance test. The statistical software package SPSS Statistics v.25 (IBM, Chicago, IL, USA) was used for all analyses; p ≤ 0.05 was considered statistically significant. When an overall significant difference was detected, preplanned pairwise comparisons of injured versus repaired and injured versus meniscectomy groups were carried out using a Wilcoxon signed-rank test with Bonferroni correction to account for multiple variations (corrected statistical significance p ≤ 0.025).

The group size was selected based on the normalized peak pressure obtained at the lateral compartment for the first three specimens tested, as clinically relevant differences in the contact parameters are unknown. Calculations using G*Power 3.1.9.2 software^29^ yielded a minimum group size of n = 7 with the Wilcoxon signed-rank test for a computed effect size of 1.5 between the injured and intact conditions at α = 0.05 with a power of 0.8, and n = 4 for a computed effect size of 2.1 between treatment conditions at each flexion angle tested using Friedman’s test. Thus, a conservative sample size of n = 9 was initially chosen, which is in accordance with prior studies in cadaveric knees^1,3,4,21,27,30^. One specimen was discarded due to anatomical abnormalities, leaving the final sample size n = 8. A sensitivity power analysis with n = 8, α = 0.05, and (1-β) = 0.8 showed a minimum detectable effect of 1.2 for the planned paired comparisons using the Wilcoxon signed-rank tests.

## Results

### Normalized contact area

**Figure 4:**
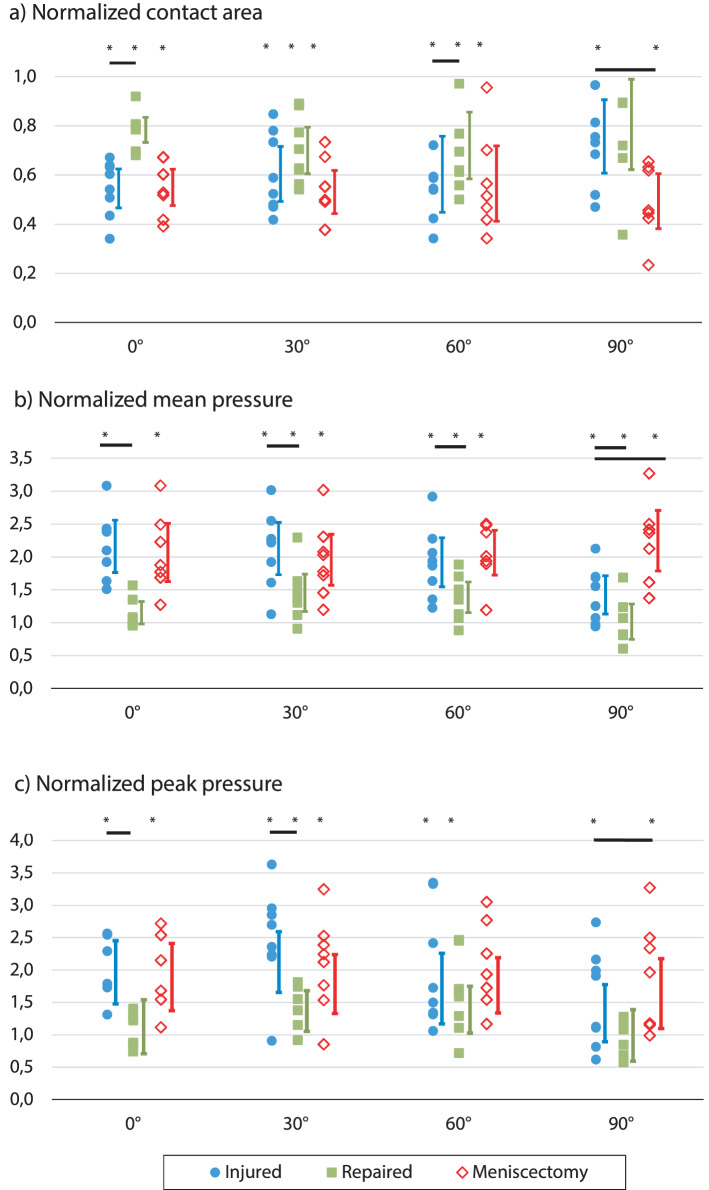
Normalized contact area (**a**), mean pressure (**b**), and maximum pressure (**c**) at the lateral compartment for the three altered meniscal conditions (injured, repaired, and total meniscectomy) for the four knee flexion angles tested; The vertical line near each dot group represents the 95% CI interval of the mean for the group data. Horizontal lines represent significant differences between groups in the preplanned pairwise comparisons. *Significant difference with respect to the intact condition.

At the compartment of the lesion (Table 1, Fig. 4a), the ARLM avulsion produced a significant decrease in the contact area relative to the control for all flexion angles (p = 0.012 at 0°, p = 0.012 at 30°, p = 0.017 at 60°, and p = 0.025 at 90°); the effect was more pronounced at full extension, with a reduction of the mean contact area by 45%, while it was the lowest at 90° flexion, reaching only a 24%. Meniscectomy also showed significant differences relative to the control at all angles (p = 0.012 at 0°, p = 0.012 at 30°, p = 0.018 at 60°, p = 0.018 at 90°), the differences were of a similar magnitude to those caused by ARLM avulsion at low flexion but were higher at 90° (p = 0.018) when the effect of anterior root detachment is less pronounced.

**Table 1:** Normalized contact area relative to the intact condition in the lateral and medial compartments at each flexion angle for the 3 altered meniscal conditions (given as mean value with the 95% CI in parentheses).

	0°	30°	60°	90°
**Lateral**				
Intact (mm^2^)	58.39 (53.86, 62.92)	53.90 (45.15, 62.64)	49.65 (38.04, 61.26)	38.89 (31.22, 46.55)
**Normalized area**				
Injured	0.55 (0.48, 0.62)*	0.60 (0.49, 0.73)*	0.60 (0.45, 0.76)*	0.76 (0.61, 0.91)*^†^
Repaired	0.77 (0.71, 0.83)*^•^	0.70 (0.61, 0.80)*	0.72 (0.59, 0.86)*^•^	0.81 (0.62, 0.99)
Meniscectomy	0.54 (0.46, 0.61)*	0.53 (0.44, 0.62)*	0.57 (0.41, 0.72)*	0.49 (0.38, 0.61)*^•^
**Medial**				
Intact (mm^2^)	54.23 (43.97, 64.62)	43.18 (33.82, 52.54)	43.86 (36.66, 51.06)	43.93 (37.81, 50.05)
**Normalized area**				
Injured	0.90 (0.72, 1.07)	0.77 (0.57, 0.97)	0.87 (0.66, 1.84)	0.95 (0.76, 1.13)
Repaired	1.03 (0.95, 1.12)	0.96 (0.84, 1.09)	0.78 (0.93, 1.08)	1.02 (0.78, 1.25)
Meniscectomy	0.89 (0.76, 1.02)	0.82 (0.70, 0.92)	0.85 (0.71, 1.00)	0.91 (0.76, 1.05)

Values of contact area (mm^2^) in the Intact group are provided for reference.

*Significant difference with respect to the intact condition.

^†^Significant difference with respect to meniscectomy.

^•^Significant difference with respect to injured.

The in-situ repair was unable to completely recover the lateral contact area, which was significantly different from the intact group for flexion angles of 0°(p = 0.012), 30°(p = 0.012), and 60°(p = 0.025), and it showed a tendency to significance at 90°(p = 0.063). However, the repair did show a partial recovery with respect to the lesion as the contact area was closer to the intact condition at all flexion angles, showing an increase in the mean contact area from injured to repaired by 40% at extension, 17% at 30°, 20% at 60°, and 7% at 90°. Said recovery reached significance at 0°(p = 0.024) and 60°(p = 0.05).

In the medial condyle (Table 1), no significant differences were detected in terms of contact area at any flexion angle between the meniscal conditions tested.

### Normalized mean pressure

In the lateral condyle (Table 2, Fig. 4b), the injury produced a significant increase in the mean pressure at all flexion angles (p = 0.018 at 0°, p = 0.012 at 30°, p = 0.012 at 60°, p = 0.036). The effect was more pronounced at extension and softened as flexion progressed, with an increase of 115% at full extension versus 41% at 90°. Meniscectomy also showed significant differences with respect to intact condition at all angles (p = 0.018 at 0°, p = 0.012 at 30°, p = 0.018 at 60°, p = 0.018 at 90°), although without showing such increase of the alteration with flexion. Moreover, compared to the avulsion, meniscectomy only showed significant differences at 90° (p = 0.018).

**Table 2:** Normalized mean pressure relative to the intact condition in the lateral and medial compartments at each flexion angle for the 3 altered meniscal conditions (given as mean value with the 95% CI in parentheses).

	0°	30°	60°	90°
**Lateral**				
Intact (N/mm^2^)	1.02 (0.80, 1.24)	1.06 (0.77, 1.34)	1.17 (0.78, 1.56)	1.26 (1.07, 1.45)
**Normalized pressure**				
Injured	2.15 (1.75, 2.55)*	2.12 (1.72, 2.52)*	1.91 (1.54, 2.28)*	1.41 (1.12, 1.70)* *†*
Repaired	1.14 (0.96, 1.31)^•^	1.44 (1.16, 1.73)*^•^	1.38 (1.14, 1.61)*^•^	1.01 (0.74, 1.27)^•^
Meniscectomy	2.06 (1.62, 2.50)*	1.95 (1.56, 2.33)*	2.05 (1.71, 2.40)*	2.24 (1.78, 2.70)*^•^
**Medial**				
Intact (N/mm^2^)	0.79 (0.43, 1.15)	1.91 (1.01, 1.61)	1.39 (1.02, 1.77)	1.41 (1.00, 1.83)
**Normalized pressure**				
Injured	1.23 (0.97, 1.58)	1.24 (0.84, 1,64)	1.16 (0.88, 1.44)	1.09 (0.93, 1.35)
Repaired	1.24 (0.96, 1,52)	1.18 (0.93, 1,43)	1.18 (0.93, 1.44)	1.27 (0.87, 1.31)
Meniscectomy	1.22 (0.84, 1.60)	1.39 (1.11, 1.68)	1.17 (0.91, 1.42)	1.16 (0.96, 1.36)

Values of mean pressure (N/mm^2^) in the Intact group are provided for reference.

*Significant difference with respect to the intact condition.

^†^Significant difference with respect to meniscectomy.

^•^Significant difference with respect to injured.

The repair of the root decreased the mean pressure compared with the injury at all flexion angles, achieving a reduction by 47% at extension, 32% at 30°, 28% at 60°, and 28% at 90°. The difference was statistically significant at all angles (p = 0.018 at 0°, p = 0.017 at 30°, p = 0.012 at 60°, and p = 0.018 at 90°). In terms of this parameter, the values achieved with the repair were different from those of the intact condition at flexion angles of 30° (p = 0.017) and 60° (p = 0.12).

In the medial condyle (Table 2), no statistical differences between tested conditions were detected.

### Normalized peak pressure

In the lateral condyle (Table 3, Fig. 4c), the injury produced a significant increase in the peak pressure compared to the intact group for all flexion angles except 90° (p = 0.018 at 0°, p = 0.017 at 30°, and p = 0.012 at 60°). The alteration was again more pronounced in extension than at high flexion. As for previous parameters, meniscectomy significantly altered peak pressure (p = 0.018 at 0°, p = 0.017 at 30°, p = 0.018 at 60°, p = 0.028 at 90°) but without a particularly greater influence at low flexion. Furthermore, significant differences between the injured and meniscectomy groups were found only at 90° (p = 0.018).

**Table 3:** Normalized peak pressure relative to the intact condition in the lateral and medial compartments at each flexion angle for the 3 altered meniscal conditions (given as mean value with the 95% CI in parentheses).

	0°	30°	60°	90°
**Lateral**				
Intact (N/mm^2^)	2.91 (2.18, 3.65)	3.53 (1.98, 5.07)	3.67 (2.45, 4.89)	4.35 (3.87, 4.84)
**Normalized pressure**				
Injured	2.30 (1.73, 2.87)*	2.48 (1.93, 3.03)*	2.00 (1.37, 2.64)*	1.56 (1.04, 2.08)^†^
Repaired	1.32 (0.83, 1.81)^•^	1.60 (1.23, 1.97)*^•^	1.62 (1.20, 2.05)*	1.16 (0.69, 1.63)
Meniscectomy	2.21 (1.61, 2.82)*	2.09 (1.56, 2.62)*	2.06 (1.57, 2.56)	1.91 (1.28, 2.54)*^•^
**Medial**				
Intact (N/mm^2^)	2.91 (1.66, 4.17)	5.58 (3.54, 7.62)	5.11 (3.35, 6.88)	6.41 (4.19, 8.63)
**Normalized pressure**				
Injured	1.21 (0.84, 1.58)	0.99 (0.65, 1.32)	1.07 (0.85, 1.30)	0.99 (0.76, 1.21)
Repaired	1.21 (1,04, 1.39)	0.92 (0.68, 1.16)	1.12 (0.92, 1.33)	1.27 (0.92, 1.62)
Meniscectomy	1.08 (0.84, 1.58)	1.06 (0.65, 1.32)	1.07 (0.85, 1.30)	0.97 (0.76, 1.21)

Values of peak pressure (N/mm^2^) in the Intact group are provided for reference.

*Significant difference with respect to the intact condition.

^†^Significant difference with respect to meniscectomy.

^•^Significant difference with respect to injured.

After repair, the mean normalized peak pressure decreased, reaching a level closer to the intact condition values, with significant differences found at 30° (p = 0.017) and 60° (p = 0.025) when comparing the intact and repaired groups. Decreases from the injured to the repaired condition reached 43% at extension, 35% at 30°, 19% at 60°, and 26% at 90°, with significant differences when the lesion was of more influence at 0° (p = 0.018) and 30° (p = 0.025).

In the medial condyle (Table 3), no statistical differences were found between the tested conditions.

## Discussion

The main findings of this study are that avulsion of the ARLM significantly alters the contact pressure distribution on the tibiofemoral cartilage of the compartment with the lesion; the alteration was especially pronounced at low flexion angles where it showed effects similar to meniscectomy. Surgical repair of the root using an in-situ technique partially restored the pre-injury condition.

It was confirmed that the lesion significantly reduced the contact area in comparison to the intact knee at all flexion angles tested and increased the mean and peak pressures on the cartilage of the injured compartment at flexion angles between 0° and 60°. Such alterations were more acute at full extension than at greater flexion angles. Even more, the lesion resembles a total lateral meniscectomy except at 90°, where the effects of the lesion were less pronounced. Specifically, changes in the mean values at full extension multiplied those observed at 90° by a factor of 1.4 for contact area, and 1.5 for mean and peak pressures. On repairing, all contact parameters showed a recovery towards the levels of the intact condition, especially at low flexion angles. However, most values reached were still statistically different from the intact condition, thus showing that the recovery was incomplete.

Most biomechanical studies on meniscal root avulsion focus on the posterior roots. Evidence of the alteration of contact pressures caused by this lesion of medial^1,4,31,32^ and lateral^3,6,22,23,30,32,33^ menisci have been published. The alterations reported after detachment of the posterior root of the medial meniscus are of similar magnitude to our findings in terms of mean contact area, peak and mean pressure in the injured compartment, although there is no a clear range of knee flexion most influenced by the injury accordling the published data. Focusing on the lateral meniscus, the detachment of the posterior root significantly reduces the contact area and increases the mean and peak pressures on the injured compartment^3,6,21,22,30^, analogous to what we found for anterior root avulsion. The variations from the intact condition reported with a detachment of the posterior root by LaPrade et al.^3^, when pooled across all angles (34% in contact area, 56% in mean and peak pressures) show magnitudes that are similar in the contact area and less pronounced in terms of pressures than those found in our work with the anterior root avulsion (37% in contact area, 90% in mean pressure and 109% in peak pressure). This result suggests that, when not repaired, the anterior root avulsion can lead to cartilage damage comparable to a detached posterior root.

On the other hand, when the biomechanical consequences of the posterior root avulsion are assessed in a range of flexion angles^3,6,33^, the intensities of alterations of the contact parameters are greater at the higher flexion angles. Laprade et al.^3^ reported a variation relative to intact in the contact area that was 1.5 greater at 90° than at full extension, 1.9 in mean pressure, and 1.8 in peak pressure. Pérez-Blanca et al.^6^ also found variations greater at 90° compared to 0°, although differences were more moderated, with factors of 1.3 for contact area and mean pressure and 1.1 for peak pressure. Recently, Ohori et al.^33^ also reported a rise in the alterations caused by the complete rupture of the posterior root as the flexion angle increased from 30 to 120, although the study was performed in a porcine model. This outcome is the opposite of our findings for the anterior root avulsion, which we believe could be owing to the articular kinematics: at low flexion angles, the contact area^34^ is placed more anteriorly, closer to the anterior root, and it is displaced posteriorly as the knee is flexed, nearing the posterior root and, therefore, it is reasonable that its detachment becomes of less influence. It is also in accordance with the conclusions of previous works that reported higher pressure load at the anterior portion of the meniscus in extension and at the posterior portion in deep flexion under compressive knee load^33,35^. Therefore, we believe that the ARLM avulsion may be of higher clinical significance than posterior root detachment, considering that daily routine and sports activities involve longer periods of knee loading at lower flexion angles comparatively. Furthermore, it should be considered that while the stabilizing function of the posterior lateral root is reinforced by the meniscofemoral ligament (when present)^2,5,30^, there is no similar structure that collaborates with the ARLM and, therefore, its integrity may be more critical.

Regarding the success in repairing the injury, several analysis of clinical outcomes after posterior meniscal root repairs^36,37^ suggested that this intervention could retard the progression of degenerative changes in the knee in up to 80 to 84% of the patients. From a biomechanical point of view, it was reported that the repair of the posterior root using transtibial techniques partially restored the preinjury contact condition^3,6^. In our study, using an in-situ surgical technique, we also found that the repair of the ARLM partially recovered the intact condition at all flexion angles, although direct comparison of the levels of recovery is difficult due to the different surgical techniques applied which may have lead to distinct results. With the in-situ repair, the contact parameters did not reach the levels of the intact knee, with differences of up to 23% in contact area, 42% in mean pressure and 60% in peak pressure at low flexion angles. From a clinical point of view, it is not known what degree of alteration is needed to trigger the onset of knee osteoarthritis. As the in-situ repair technique applied in our research achieved a partial restoration, it could be expected to contribute in lessening knee damage as seen with the repair of posterior roots. However, the important differences observed between the contact parameters in the natural knee and after repair indicate that, application of other surgical techniques should be further investigated in order to improve the outcomes, such as root suturing or a transosseous pullout procedure.

To our knowledge, only one published study^37^ addressed the possible alterations in the contact parameters due to a lesion at the anterior area of the lateral human meniscus, although it focused on a 2 cm longitudinal tear in the peripheral 1/3 of the anterior horn, its repair, and a partial meniscectomy about the tear. Eight human knees were tested at extension and 30° flexion, subjected to an axial load similar to the compression in our study. Only partial meniscectomy showed a significant increase in the peak pressure and contact area in the injured compartment with respect to the intact knee, but neither did the tear nor its repair. In our work, we did find significant differences in the contact parameters between the intact and injury groups, which we think is due to the fact that the root avulsion analyzed in our work fully disrupt the continuity of the circumferential fiber, while the tear studied by Prince et al.^38^ does not. In line with this result, previous studies reported that incomplete radial tears of the lateral meniscus of up to 66% width in a porcine model^39^ and up to 75% width in a human model^24^ did not induce any significant changes in the contact or kinematic parameters of the knee under compression, whilst complete radial tears generated significant alterations in both cases. As for meniscectomy, we also observed changes in all contact parameters at 0° and 30°, albeit the contrast of the results is prevented because they conducted only a partial meniscectomy that preserved the continuity of the circumferential fibers.

The present work has certain limitations, owing to the use of cadaveric specimens that do not allow reproduction of the biological response of the tissues, which is inherent to the use of ex vivo specimens. As no muscle activity was reproduced, to keep the knee stable at imposed angles in high flexion conditions, the actuation necessary was supplied by blocking anteroposterior displacement, a common practice^24^ but which may have altered the final contact position at 90°. To lessen this possible effect, the flexed knee was free to reorient naturally under a load of up to 100 N before fixing this degree of freedom, following the same protocol in all meniscal conditions, and hence, we believe that the comparative result presented would stand. Additionally, no dynamic phenomena were assessed, like creep effects due to the viscoelastic response of soft tissues or cyclic loading; as in similar studies, a static compressive load was applied and the variables were registered after specimen stabilization. Also, although an arthrotomy was conducted and most of the soft tissue of the knee had to be removed and the coronal ligament partially sectioned to allow insertion of the sensors, special care was taken not to damage the meniscal roots or the knee ligaments to minimize the anatomical alterations of the joint. Finally, it has been reported that the load output of the Tekscan pressure sensors used to measure intraarticular pressures on cadaveric specimens could diminish over time and after the application of several dynamic loading cycles^26–28^. To correct this possible inaccuracy, the pressure measured in each test was normalized by the total applied force.

In conclusion, avulsion of the ARLM produces significant alterations in the contact biomechanics of the knee, increasing the pressure and reducing the contact area on the articular cartilage of the injured compartment. Alterations were greater at low knee flexion angles, where they were similar to total meniscectomy. In-situ repair partially restored these biomechanical alterations to the pre-injury condition.
